# Teacher Support Matters: The Effect of Self-Control Demands on Safety Behavior of Vocational High School Students in China

**DOI:** 10.3390/ijerph19127220

**Published:** 2022-06-13

**Authors:** Xiao Yuan, Yongjuan Li, Yaoshan Xu, Huifang Yang

**Affiliations:** 1 School of Economics and Business Administration, Central China Normal University, Wuhan 430079, China; yuanx@ccnu.edu.cn (X.Y.); yanghf@mails.ccnu.edu.cn (H.Y.); 2 CAS Key Laboratory of Behavioral Science, Institute of Psychology, Chinese Academy of Sciences, Beijing 100101, China; liyj@psych.ac.cn

**Keywords:** self-control demands, ego depletion, safety behavior, vocational student

## Abstract

The safety behavior of vocational school students is worth noting. The current study is aimed to examine the effect of self-control demands on safety behavior. Drawing on the Self-Control Resource Model, we predict that self-control demands have a negative effect on safety behavior through ego depletion and perceived teacher support moderates the link among self-control demands, ego depletion, and safety behavior. A two-wave survey was conducted and 285 vocational students participated in our study. Mediation and moderated mediation modeling analyses were carried out. Results showed that ego depletion fully mediated the link between self-control demands and safety behavior. Moreover, perceived teacher support moderated the relationship between self-control demands, ego depletion and safety behavior; for students who perceived high levels of teacher support, the negative effect of self-control demands on safety behavior via ego depletion was insignificant, while for students who perceived low levels of teacher support, their negative effect was significant. The present study clarifies the effects of self-control demands on safety behavior through the resource depletion process and highlights the importance of teacher support in buffering the negative effect of self-control demands on workplace safety. Enhancing safety management, engaging in a resource recovery activity, and providing teacher support training are effective ways to maintain high levels of workplace safety.

## 1. Introduction

Occupational health and safety is still an ever-growing issue in the worldwide context. Each year, there are over 2.3 million fatalities resulting from about 300 million work accidents [1]. Moreover, among accident-prone employees, young workers, especially those ranging from 15 to 24 years of age, suffer work-related injuries at a much higher rate because of their insufficient experience, less skill, and underestimation of workplace risk [2,3,4]. Given the high rates of occupational injury among young workers, it is necessary to emphasize the importance of developing good safety habits and exerting correct safety behaviors when they are learning technical skills in school [2]. Therefore, investigating the factors that influence the safety behaviors of vocational school students may shed some light on accident prevention in the workplace. 

Vocational school students’ safety behavior is worth noting for several reasons. First, vocational school students, different from academic high school students who continue to pursue tertiary education, are usually prepared to step out of the classroom and enter directly into the working field. This characteristic requires the vocational education system to provide necessary vocational training programs for their future careers [5]. During the vocational training period, students are exposed to the same occupational risks as workers and have a high incidence of injuries [6]. Consequently, occupational health and safety is a big issue in this group. Second, because of their underestimation of the risk of injury [4] and insufficient experience to deal with various demands [3], the youth usually do not follow the safety procedures and engage in unsafe behaviors, which in turn are more likely to result in safety accidents. Worse, safety procedures that students have learnt during the training period set the foundation for their work experiences in the future [5], which means they also may not follow the safety rules in the workplace. Therefore, it is necessary to focus on students’ safety behavior performed during their training tasks.

Current research on vocational training has focused on the effects of safety training in knowing and using this training in the workplace [2] but does not address why some students engage in more safety behaviors than others within the same training group. The exertion of safety behaviors relies on sufficient self-control resources within the individual [7,8]. Investigating whether and how vocational students allocate their self-control resources in a safety-related procedure might provide answers to the question of why some students perform more safely within the same group.

**Figure 1:**
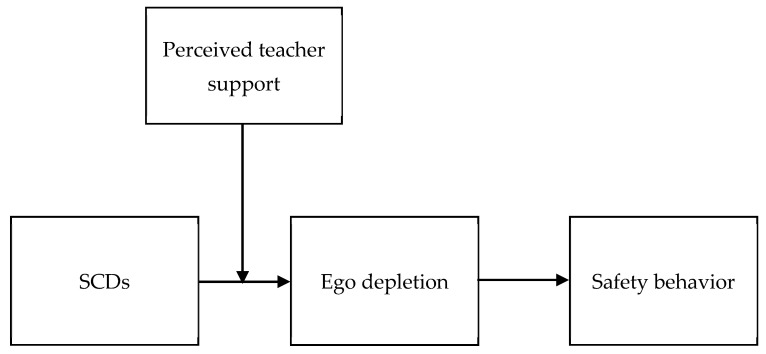
The hypothesized model.

Schmidt and Neubach (2007) [9] coined the term “self-control demands (SCDs)” to characterize those demands that would consume individuals’ self-control resources. SCDs are labeled as those demands which require inhibiting spontaneous responses and controlling affective states, resisting distractions caused by irrelevant tasks, and overcoming the lack of motivation resulting from boring tasks [10]. Drawing on the Self-Control Resource Model, we considered that SCDs may impair the safety behavior of vocational students through self-control resource depletion. Consequently, the main purpose of the present study was to explore whether self-control demands would trigger ego depletion and then reduce vocational students’ safety behavior. Given the potentially negative effect of self-control demands on safety behaviors, examining the factors that may buffer their negative relationships is also crucial. The role of the teacher in caring about student safety and instructing them to perform safely is particularly important and should be recognized [2]. Thus, we considered teacher support a critical moderator. Figure 1 shows the hypothesized model of this study.

### 1.1. Adverse Effect of SCDs on Safety Behavior: Ego Depletion as the Mediator

A series of research on self-control has demonstrated that exercising self-control leads to cognitive and behavioral control deficits [7,8,9,10]. As the increasing importance of self-control in the workplace has been recognized, examining the role of work-related SCDs has become a major issue. According to the Self-Control Resource Model, self-control resources are limited general resources, and any kinds of self-control acts (e.g., emotional control and thought suppression) deplete these common resources [11]. Consequently, confronted with tasks that need long-time concentration, employees deplete their limited resources by trying to ignore task-irrelevant stimuli [12] and overcome inner resistances [13], as well as to suppress inappropriate thoughts and emotions [9]. Such self-control efforts elicit a reduction in self-control resources, and the state of depletion after consuming self-control resources is known as ego depletion, which is characterized by low willpower, or low ability for further self-control [14]. Therefore, individuals who perceive ego-deletion allocate few resources for subsequent self-control tasks [11]. In line with this argument, empirical research has shown that employees who have responded to SCDs are more likely to report work burnout and impaired well-being [15].

In the workplace, safety behavior includes safety compliance behavior (i.e., rule-complying behavior) and safety participation behavior (i.e., voluntary safety-related behaviors) [8]. As individuals who experience ego depletion have a diminished ability to perform subsequent self-control activities [11], we propose such students will have an impaired ability to inhibit unsafe behavioral impulses. As noted above, students who suffer from ego depletion will have difficulty with concentration and vigilance [16]. Thus, in performing safety-related tasks, they are more likely to be disturbed by irrelevant stimuli and have decreased alertness, which makes them less likely to identify potential hazards in time [17]. Beyond the possible effect of cognitive deficits, depleted individuals cannot control their negative emotions and these uncontrollable negative emotions also damage individual safety behavior [18,19]. Additionally, ego-depleted individuals find it difficult to overcome impulses and resist temptations and tend to choose immediate interests rather than long-term goals [20,21]. Specific to the safety domain, unsafe behaviors such as taking shortcuts may make work more efficient, which would bring more performance reward but have a longer-term cost of decreasing safety. Individuals in a state of ego depletion may choose the immediate benefits and perform unsafely. Consistent with our argument, an increasing amount of research has indicated that executing safety behaviors relies on sufficient self-control resources [7,8,22].

Considering the established relationship between SCDs and ego depletion and the relationship between ego depletion and safety behavior, we argue that SCDs have a negative effect on safety behavior through ego depletion. As such, we propose the following hypothesis:

**Hypothesis** **1.** *Ego depletion mediates the relationship between SCDs and safety behavior.*

### 1.2. Buffering Effect of Perceived Teacher Support on the SCDs–Ego Depletion Relationship

In line with the Self-Control Resource Model, the psychological costs of SCDs are considered to be the result of the repeated depletion of limited self-control resources [14]. Consequently, in cases of high SCDs and associated states of ego depletion, the replenishment of resources can be expected to prevent further depletion. Since social support is identified as a vital resource for helping employees cope with several work demands [23], we argue that perceived teacher support plays a significant role in buffering the effect of SCDs on ego depletion for vocational students in school settings. 

Perceived teacher support is viewed as a student’s perception of general support or specific supportive behaviors from a teacher in their social network, including emotional support, instrumental support, informational support, and appraisal support [24]. Perceiving high levels of teacher support, students feel cared for, loved, and trusted (i.e., emotional support), which increases their positive emotion and decreases negative emotion [24]. Since teacher support helps to reduce negative emotion, students use few self-control resources to control negative emotion; additionally, positive emotions could also help people regain their self-control resources [25]. In this case, students who perceived high levels of teacher support experience less ego depletion than those who perceived low levels of teacher support. Moreover, when students encounter SCDs such as distractions or unsafety behavior habits in their training courses, supportive teachers will spend time to teach them safety-related knowledge (i.e., instrumental support), provide certain guidance to help them resist distractions, and encourage them to follow the correct training procedures instead of bad habits (i.e., informational support) [24]. Therefore, students aided by supportive teachers use few self-control resources to deal with those SCDs and reduce their depletion levels. In addition, supportive teachers give students positive feedback when they exert safety procedures (i.e., appraisal support). As a result, vocational students perceive safety as a high priority in their training programs and are likely to allocate enough self-control resources to safety-related tasks [7]. As such, we consider that perceived teacher support will buffer the positive effect of SCDs on ego depletion and propose the following hypothesis:

**Hypothesis** **2.** *Perceived teacher support moderates the relationship between SCDs and ego depletion such that the effect of SCDs on ego depletion is weaker for students who perceive high levels of teacher support compared to those who perceive low levels of teacher support.*

Furthermore, we propose that SCDs lead to students’ ego depletion, which in turn can decrease their safety behaviors. Therefore, confronted with various SCDs, students perceiving high levels of teacher support deplete fewer resources than those perceiving low levels of teacher support; as a result, they can allocate more self-control resources on safety-related tasks and maintain higher levels of safety behaviors. Following this line of reasoning, we argue that perceived teacher support buffers the negative effects of SCDs on safety behaviors by reducing the degree of ego depletion. Accordingly, we propose the potential moderated mediation hypothesis as follows:

**Hypothesis** **3.** *The mediated relationships among SCDs, ego depletion, and safety behaviors are weaker for students who perceive high levels of teacher support than those who perceive low levels of teacher support.*

## 2. Materials and Methods

### 2.1. Participants and Procedures

Our sample was students from a vocational high school whose employment positions were electric train drivers and vehicle maintenance workers in China. They had been studying at the school for a year, including theoretical and practical courses. The theoretical courses were knowledge-based lectures, while the practical courses focused on specific operational training. Although their practical training courses were conducted on simulators, their operating environment was exactly the same as the real working environment. For example, for electric train driver students, their training course was conducted on a simulator exactly like a real electric train, where they could learn the techniques of how to drive a train safely. 

Before beginning the study, students were given an informed consent form that assured confidentiality and the ability to withdraw from the study at any time. If they agreed to participate in our study, they could fill out the questionnaire.

After this instruction, one of our researchers sent two separate paper-and-pencil questionnaires to potential respondents, spaced two weeks apart from each other in order to reduce the common method biases in a cross-sectional study [26,27]. At time 1, we sent 300 questionnaires, which included a measure of SCDs the participants experienced, a measure of perceived teacher support, and a demographics questionnaire, to potential respondents, and 294 were returned. At time 2, questionnaires that included a measure of ego depletion and a measure of safety behavior were only sent to the 294 respondents who had completed the first survey, and 285 were returned. We performed an independent sample t-test and chi-squared test between those who only completed the first survey and those who completed both surveys. Results showed no significant differences in gender (χ^2^(1) < 0.001, ns.), age, scores on SCDs the first time, and perceived teacher support the first time (ts < 0.738, ns.), which indicated that no response bias existed. Therefore, the 285 respondents comprised the final sample. Due to the specificity of the profession, 253 of them were male and only 32 were female. The age of participants ranged from 14 to 22 years old, with an average age of 16.09 years.

### 2.2. Measures

SCDs. SCDs were measured using the 15-item self-control demand scale developed by Schmidt and Neubach [28]. This measure was developed to assess the demands on workers to inhibit spontaneous and impulsive responses, to avoid distractions and avoid task-irrelevant stimuli, and to overcome the lack of motivation for dull, repetitive tasks [28]. A sample item is “In order to achieve my performance goals, I must not let myself be distracted.” Participants responded to these items on a 5-point scale ranging from 1 (strongly disagree) to 5 (strongly agree). In this scale, the higher the score, the higher the degree of SCDs.

Ego depletion. Ego depletion was assessed with the state self-control capacity scale developed by Twenge, Muraven, and Tice [29]. This 25-item scale assessed the perceptions of the momentary availability of self-control resources. To control the length of the questionnaire, we followed the suggestion of Kam et al. [30] and only included 8 items that directly assessed participants’ capacities to override or alter their behaviors in the moment. A sample item is “If I were tempted by something right now, it would be difficult to resist.” Participants were asked to provide ratings on a 5-point scale from 1 (strongly disagree) to 5 (strongly agree) and a high score indicated high levels of ego depletion.

Perceived teacher support. Using the teacher support subscale of the Child and Adolescent Social Support Scale developed by Malecki and Demaray, perceived teacher support was measured with 10 items, which assessed students’ perceived concern, instruction, and positive feedback from teachers [24]. A sample item is “My teacher cares about me.” Participants responded on a 6-point scale ranging from 1 (never) to 6 (always). A high score indicated a high level of perceived teacher support. 

Safety behaviors. Safety behaviors were measured using a 6-item scale developed by Neal and Griffin, which consisted of two dimensions: safety compliance and safety participation [31]. This measurement assessed the frequency of students’ safety behaviors during safety training. Participants were instructed to rate the frequency of their safety behaviors using a 7-point scale ranging from 1 (never) to 7 (frequently). An example of a safety compliance item is “I use all the necessary safety equipment at work.” An example of a safety participation item is “I promote the safety program within the organization.” A high score indicated a high level of safety behaviors.

Control variables. Demographic variables such as gender and age were controlled to avoid potential confounding effects on the dependent variables.

### 2.3. Data Analysis

In our study, a confirmatory factor analysis was first conducted to determine whether our constructs had satisfactory discriminant validity by using IBM SPSS AMOS 26 software (IBM, Armonk, NY, USA). Then, the descriptive statistics, coefficient alphas of scales, were reported and correlation analysis and regression analysis were performed by using IBM SPSS Statistics 22.0 statistical software. Finally, the hypothesized mediating effect and moderated mediating effect were examined by using PROCESS developed by Hayes [32], which is a plug-in program in SPSS software.

## 3. Results

### 3.1. Preliminary Analyses

We conducted a four-factor model (i.e., SCDs, ego depletion, perceived teacher support, and safety behaviors) of confirmatory factor analysis. Confirmatory factor analysis results showed that χ^2^/df was 1.93, the root mean square error of approximation (RMSEA) was 0.06, the comparative fit index (CFI) was 0.91, and the Tucker–Lewis index (TLI) was 0.90, which suggested that the four-factor model had acceptable fit.

### 3.2. Descriptive Statistics and Correlations

Table 1 shows the mean, standard deviation, coefficient alpha, range, and correlation coefficient of each variable. As can be seen, SCDs had a significant positive correlation with ego depletion, and ego depletion had a negative correlation with safety behavior, which was consistent with our hypothesis. We also found that perceived teacher support had a positive correlation with safety behavior, which implied that teacher support played an important role in improving students’ safety behavior. In addition, we found a significant positive correlation between age and safety behavior, indicating that the older the students were, the more safety behavior they had. We also compared the gender differences in SCDs, ego depletion, perceived teacher support, and safety behavior, and the results showed there was no gender difference in these four variables (ts < 1.515, ns.). 

### 3.3. Hypothesis Testing

To reduce the effect of multicollinearity, we first standardized the independent variable and moderator variable and then calculated interactions based on their standardized values [33]. Similarly, we also standardized outcome variables to control effect sizes [8]. After that, hierarchical regression analysis was adopted to initially test potential effects. We further used Model 4 in PROCESS to examine the significance of indirect effect and Model 7 in PROCESS to examine potential conditional indirect effects under the difference levels of perceived teacher support, respectively [34]. 

Table 2 presents the hierarchical regression analysis result. As shown in Table 2, SCDs were positively correlated with ego depletion (B = 0.18, *p* < 0.01), while ego depletion was negatively correlated with safety behavior (B = −0.20, *p* < 0.001), which suggested that SCDs may negatively affect safety behavior through ego depletion. We used Model 4 in the PROCESS program to estimate the potential indirect effects. The unstandardized coefficients of the indirect effects and 95% confidence intervals around the estimation of the indirect effects using bootstrapping procedures with 1000 resamples are reported here [32]. As suggested by Hayes (2013), if the 95% confidence interval includes zero, the indirect effect is insignificant; if zero is not included in the 95% confidence interval, we can conclude the indirect effect is significant. The results showed that the coefficient for the indirect effects of SCDs on safety behavior through ego depletion was −0.035 with a 95% confidence interval between −0.081 and −0.008, which does not include zero. This result indicated that the indirect effect of SCDs on safety behavior through ego depletion was significant, which supports Hypothesis 1.

**Figure 2:**
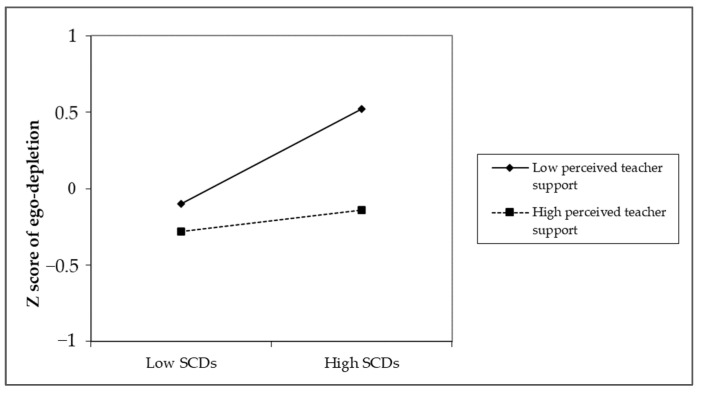
The interaction effect of SCDs and teacher support on ego depletion.

In support of our prediction that perceived teacher support would buffer the effects of SCDs on ego depletion, we detected an interaction effect in our regression analysis. As shown in Table 2, the interaction effect between SCDs and perceived teacher support on ego depletion was significant (B = −0.12, *p* < 0.05), suggesting that perceived teacher support could reduce the ego depletion degree which was caused by SCDs. To further probe the potential effect of SCDs on ego depletion under different levels of perceived teacher support, we conducted the simple slope test. The result showed that, for employees who perceived high levels of teacher support (1 SD above the mean), SCDs were insignificant in predicting ego depletion (B = 0.07, *p* > 0.05); further, for those who perceived low levels of teacher support (1 SD below the mean), SCDs significantly positively predicted ego depletion (B = 0.31, *p* < 0.001). These findings support Hypothesis 2. Figure 2 illustrates the effect of SCDs on ego depletion under high and low levels of perceived teacher support, respectively.

To test the potential moderated mediation hypothesis that perceived teacher support would attenuate the indirect negative effect of SCDs on safety behavior through ego depletion, we used Model 7 in SPSS PROCESS [32]. Specifically, the estimated coefficients of the conditional indirect effects of SCDs on safety behaviors through ego depletion at high and low levels of perceived teacher support are reported. The results demonstrate that, under high levels of teacher support, the indirect effect of SCDs on safety behavior through ego depletion was insignificant (coefficient = −0.014; 95% confidence interval ranged from −0.060 to 0.016); whereas under low levels of teacher support, the indirect effect of SCDs on safety behavior through ego depletion was significant (coefficient = −0.060; 95% confidence interval ranged from −0.120 to −0.016). Moreover, the index of moderated mediation was 0.023 with a 95% confidence interval between 0.002 and 0.057, which did not include zero, indicating that the moderated mediation effect was significant. In addition, we adopted the suggestion of Edwards and Lambert to compare their indirect effects under high and low levels of perceived teacher support [35]. The results showed that there was a significant difference in the indirect effects of SCDs on safety behavior through ego depletion (the difference was 0.046, *p* < 0.05). These findings support Hypothesis 3. 

## 4. Discussion

The purpose of this study was to explore the potential mechanisms of maintaining vocational students’ high levels of safety behavior. More specifically, we investigated whether SCDs affected their safety behavior. In addition, we also aimed to find out whether perceived teacher support could be considered a crucial resource to attenuate the negative effect of SCDs on safety behavior. The findings demonstrated that various SCDs depleted vocational students’ limited self-control resources, and students who lacked sufficient self-control resources could not ensure the correct exertion of safety behavior. SCDs decreased safety behavior via ego depletion. Furthermore, the results also indicated the moderating effect of perceived teacher support on the relationship between SCDs and ego depletion. Specifically, for students who perceived high levels of teacher support, they depleted fewer resources to deal with SCDs than those who perceived low levels of teacher support.

In terms of the mediation effect of ego depletion on the link between SCDs and safety behavior, we were more interested in the possible moderating effect of perceived teacher support in attenuating the negative indirect effect of SCDs on safety behavior. The moderated mediation analysis result suggested that for students under low levels of teacher support, the indirect effect was stronger. In contrast, for students under high levels of teacher support, the indirect effect was weaker and became insignificant. This promising result suggested that SCDs and ego depletion may not necessarily reduce safety behavior.

### 4.1. Theoretical Implications 

This study contributes to the literature on SCDs and safety behavior. A number of studies considered SCDs as sources of stress at work and explored the potential effect of SCDs on psychological strain and unhealthy behavior (e.g., alcohol intake) in the occupational health domain [36,37]. We extended the research on SCDs, using a sample of vocational students, to examine their relationship with safety behavior in electric train driving and vehicle maintenance training programs. For vocational students whose employment positions are safety-critical, performing safely is recognized as an important guarantee in preventing accidents [38]. In the present study, we were able to determine the factors that might influence students’ safety behavior, such as SCDs. 

Moreover, we explored the relationship between SCDs and the safety behavior of vocational students from the perspective of resource depletion. Based on the Self-Control Resource Model, the mechanism of self-control resource depletion contributes to understanding the occurrence of organized deviant behavior [39,40]. In our study, noncompliance with safety procedures can be regarded as an organized deviant behavior. Consequently, for vocational students who work in safety-critical positions, they must allocate enough self-control resources to ensure workplace safety [7,22]. However, various SCDs deplete their limited resources to cope with negative emotions or distractions, leading to ego depletion. Under these circumstances, students lack enough self-control resources to resist unsafe work behavior.

Furthermore, previous research has evidenced that the response to SCDs varies from person to person, and SCDs do not always lead to burnout or other negative work outcomes [10,15]. In our study, we found that teacher support moderated the link among SCDs, ego depletion, and safety behavior with a sample of vocational students in China. Since students from collectivist cultures are more likely to obey instructions, teacher support may play a more prominent role in buffering the negative impact of SCD on safe behavior. During the training program, supportive teachers would express their care and unconditional positive regard to students, spend time to explain safety knowledge and safety procedures to them, give time to a detailed instruction about safety operation, and provide positive feedback when they exerted safety procedures. Perceiving high levels of teacher support, vocational students would perceive safety as a high priority in their training program and were likely to allocate more self-control resources to safety-related tasks. In addition, because students were expected to obey teachers’ demands, the safety compliance behavior may have made up the decreasing safety participation behavior, resulting in a high level of safety behavior. When safety behavior is internalized into safety habits, it will help to maintain high levels of safety behavior in the work environment, which is away from teachers. This finding strengthens our understanding of the moderating effect of perceived teacher support and reveals a promising result that SCDs and ego depletion are not always detrimental to safety behavior.

### 4.2. Practical Implications

Data from a survey on fatal occupational injuries in China have shown that young workers have a higher injury rate than the old workers [41]. Vocational students can be included in this injury-prone group. Thus, how to maintain high levels of safety behavior in this group is important. For students in vocational school, during their training period they are exposed to the same occupational risks as workers [6]. In order to improve workplace safety, vocational schools should enhance safety management to ensure the safety of vocational students. For example, vocational students who do not wear safety equipment properly or cannot repeat the safety procedures are not allowed to operate on the simulator. Vocational students also encounter various SCDs. Because of their insufficient experience to deal with these various demands [3], they may deplete more self-control resources than those experienced employees. We must ensure that there are enough self-control resources for the vocational students to utilize. As such, after dealing with SCDs, depleted students could engage in a resource recovery activity and then participate in a safety activity to maintain high levels of safety behavior [42]. 

Our study revealed the role of teacher support in improving vocational students’ safety behavior and in attenuating the negative relationship among SCDs, ego depletion, and safety behavior. Therefore, at the school level, choosing a supportive teacher as vocational students’ training instructor could help to decrease self-control resource depletion, which in turn would improve their safety behavior. Moreover, vocational school also can develop teacher training programs including unconditional positive regard so that every teacher has the ability to provide support to students and teach them how to deal with work-related SCDs.

### 4.3. Limitations and Future Directions 

Although the moderated mediation result is significant, there are still several limitations that should be addressed in future research. First, self-reported data collection may raise concerns about common method bias. We conducted the Harman single-factor test to perform factor analysis on all variables. The results showed that only 21.96% of the variation was explained by the first principal component, which was below the critical value (40%), indicating that common method bias is not serious among the variables measured. Furthermore, although four variables were measured by using a self-reported survey, it was important to note that we measured SCDs, teacher support, ego depletion, and safety behavior at different times; collecting data at different times could reduce the common method bias [27]. Future research could use multisource data to further evidence the mechanisms of interest. It would be especially helpful to have others rate students’ use of safety procedures, as self-reported data might be the most prone to subject bias. 

Another limitation exists with regard to low explained variance, which may indicate that there is more variance to account for in this model. Future research could account more variables when validating this model. 

Furthermore, although the sample of the vocational students in safety-related majors is appropriate for our study, 285 responses from one vocational school may limit generalizability. Future research could collect more students’ responses from multiple vocational schools and multiple safety-related majors to further evidence our model and increase the generalizability of the results.

Finally, despite that we mentioned teacher support included emotional, instrumental, informational, and appraisal support and the measurement we used in our study addressed all four types, the measurement was not originally intended to produce type scores. As a result, we did not explore which type of support had the strongest moderation effect. We followed the suggestion of Malecki and Demaray [24] and regarded teacher support as a whole. Future research could further examine which support directly deals with ego depletion based on four-type measurement. 

## 5. Conclusions

The present study provides a theoretical framework and empirical support for the relationship between SCDs and workplace safety behavior drawing on the Self-Control Resource Model. SCDs can reduce vocational students’ safety behaviors through resource depletion, and perceived teacher support attenuates the negative effect of SCDs on safety behavior. Specifically, the negative indirect effect of SCDs on safety behavior through ego depletion is stronger for students who perceive low levels of teacher support. A better understanding of the moderated mediation mechanism could help vocational training schools and teachers improve vocational students’ safety behavior and maintain workplace safety.

## Figures and Tables

**Table 1:** Descriptive statistics and correlation analysis (N = 285).

Variable	α	Range	M	SD	1	2	3	4	5
1. Age	-	-	16.09	1.23	-				
2. SCDs	0.82	1–5	3.43	0.58	0.03	-			
3. Ego depletion	0.87	1–5	2.46	0.84	−0.01	0.18 **	-		
4. Perceived teacher support	0.95	1–6	3.89	1.14	−0.07	−0.00	−0.21 ***	-	
5. Safety behaviors	0.95	1–7	5.36	1.30	0.17 **	−0.02	−0.20 ***	0.241 ***	-

Note: ** *p* < 0.01, *** *p* < 0.001.

**Table 2:** Coefficient estimates for moderated mediation model for safety behaviors (N = 285).

Variable	First Stage			Second Stage		
	Ego Depletion			Safety Behaviors		
	Model 1	Model 2	Model 3	Model 4	Model 5	Model 6
Constant	0.00 (0.06)	0.00 (0.06)	0.00 (0.06)	−0.00 (0.06)	−0.00 (0.06)	−0.00 (0.06)
Gender	−0.01 (0.06)	0.02 (0.06)	0.03 (0.06)	0.09 (0.06)	0.09 (0.06)	0.09 (0.06)
Age	−0.01 (0.06)	−0.03 (0.06)	−0.02 (0.06)	0.18 ** (0.06)	0.18 ** (0.06)	0.18 (0.06)
SCDs		0.18 ** (0.06)	0.19 ** (0.06)		−0.02 (0.06)	0.01 (0.06)
Perceived teacher support		−0.21 *** (0.06)	−0.21 *** (0.06)			
SCDs × Perceived teacher support			−0.12 * (0.05)			
Ego depletion						−0.20 *** (0.06)
F	0.029	5.827 ***	5.687 ***	5.56 **	3.74 *	5.79 ***
R2	0.00	0.08	0.09	0.04	0.04	0.08
R2 Adjusted	0.00	0.06	0.08	0.03	0.03	0.06
R2 changed		0.08 ***	0.02 *		0.00	0.04 ***

Note: * *p* < 0.05, ** *p* < 0.01, *** *p* < 0.001.

## Data Availability

Data are available upon reasonable request by emailing yuanx@ccnu.edu.cn.
